# Plant X-tender: An extension of the AssemblX system for the assembly and expression of multigene constructs in plants

**DOI:** 10.1371/journal.pone.0190526

**Published:** 2018-01-04

**Authors:** Tjaša Lukan, Fabian Machens, Anna Coll, Špela Baebler, Katrin Messerschmidt, Kristina Gruden

**Affiliations:** 1 National Institute of Biology, Department of Biotechnology and Systems Biology, Ljubljana, Slovenia; 2 International Postgraduate School, Ljubljana, Slovenia; 3 University of Potsdam, Cell2Fab Research Unit, Potsdam, Germany; Instituto Butantan, BRAZIL

## Abstract

Cloning multiple DNA fragments for delivery of several genes of interest into the plant genome is one of the main technological challenges in plant synthetic biology. Despite several modular assembly methods developed in recent years, the plant biotechnology community has not widely adopted them yet, probably due to the lack of appropriate vectors and software tools. Here we present Plant X-tender, an extension of the highly efficient, scar-free and sequence-independent multigene assembly strategy AssemblX, based on overlap-depended cloning methods and rare-cutting restriction enzymes. Plant X-tender consists of a set of plant expression vectors and the protocols for most efficient cloning into the novel vector set needed for plant expression and thus introduces advantages of AssemblX into plant synthetic biology. The novel vector set covers different backbones and selection markers to allow full design flexibility. We have included *ccd*B counterselection, thereby allowing the transfer of multigene constructs into the novel vector set in a straightforward and highly efficient way. Vectors are available as empty backbones and are fully flexible regarding the orientation of expression cassettes and addition of linkers between them, if required. We optimised the assembly and subcloning protocol by testing different scar-less assembly approaches: the noncommercial SLiCE and TAR methods and the commercial Gibson assembly and NEBuilder HiFi DNA assembly kits. Plant X-tender was applicable even in combination with low efficient homemade chemically competent or electrocompetent *Escherichia coli*. We have further validated the developed procedure for plant protein expression by cloning two cassettes into the newly developed vectors and subsequently transferred them to *Nicotiana benthamiana* in a transient expression setup. Thereby we show that multigene constructs can be delivered into plant cells in a streamlined and highly efficient way. Our results will support faster introduction of synthetic biology into plant science.

## Introduction

One of the main technological obstacles in plant biotechnology is cloning for delivery of multiple DNA fragments into the plant genome [1]. Therefore, there is an increased demand for the assembly, introduction and expression of DNA encoding multiple genes, which could cope with ongoing needs, such as reconstruction of complex biochemical pathways [2,3], engineering synthetic signal transduction systems [4] and other high-impact goals in plant synthetic biology [5].

Two crucial factors are required for the development of an efficient cloning strategy in plant synthetic biology: first, a method by which the foreign coding sequence (CDS) and regulatory elements, i.e. promoter and terminator sequences, can be assembled and inserted into the plant expression vector in a highly efficient and versatile manner and second, the availability of plant expression vectors capable of holding and introducing multiple expression cassettes into the plant genome. Although a remarkable variety of plant expression plasmids has already been developed, the basic design of many of these vectors is quite restrictive and rarely permits the cloning and transfer of more than a single expression cassette into plant cells [6,7].

Due to the increasing requirement for high throughput approaches to assemble complex designs, classical restriction-based cloning became limited. Critical points are low cloning efficiency, high expenditure of time, the introduction of unwanted sequences at the junction sites and the occurrence of restriction enzyme recognition sites within expression cassettes. Although the latter drawback was overcome by exploiting the power of rare-cutters for the assembly of multiple expression cassettes by designing the pAUX series of plasmids [8] and their variations pSAT [9,10], the system is still limited by the number of different commercially available rare-cutting restriction enzymes.

To overcome the limitations of classical restriction-based cloning methods, the emergence of different recombination-dependent methods was exploited for multigene cloning applications. Since the invention of the first recombination methods based on the Cre/*loxP* recombination system (see review [11]), the number of elements that can be assembled was increased by combining this system with two rare-cutter endonucleases [12]. Another recombination-based cloning method, Invitrogen’s Gateway technology, which is based on the bacteriophage λ site-specific recombination system [13] overcomes the shortcoming of classical Cre/*loxP* recombination system, since it allows delivery of DNA fragments from entry into multiple destination vectors with high specificity and efficiency [13,14,15]. Although the upgrade of the technique into Multisite Gateway enables delivery of up to three independent transcription units into plant cells [16,17], it is still limited in its flexibility due to a small number of available *att* sites. However, with MultiRound Gateway cloning, based on multiple rounds of LR recombination reactions, it is possible to assemble up to seven expression cassettes [18,19]. Despite many benefits of Gateway-based systems, there are still several drawbacks such as the introduction of unwanted cloning scars at the junction sites and high cost, especially for large scale projects [20].

Another vector set that overcomes the drawback of type II restriction enzymes was designed by Ghareeb et al., 2016 [21]. The system, named COLORFUL-Circuit, is based on the rare-cutter *Sfi*I. Since the overhang sequences generated by this restriction enzyme can be freely modified to generate unique non-palindromic ends, this system has the potential for a high throughput approach. To date, COLORFUL-Circuit assembly enables the introduction of up to five expression cassettes and is specifically designed for studies involving fusions with fluorescent proteins.

In recent years, significant efforts were dedicated to the development of simple and efficient cloning systems for standardized assembly of genetic modules, using defined rules [22,23]. High throughput modular ligation-dependent methods, efficient for multipart assemblies for expression in plants, are Golden Gate [24] and derived strategies i.e. MoClo [25], GoldenBraid [26] and GreenGate [27]. However, disadvantages of those methods are common to conventional cloning strategies, as they are based on the use of type IIS restriction endonucleases. For those, the main disadvantage is the high occurrence of recognition sites within expression cassettes, which becomes increasingly relevant in the case of large multigene constructs.

In contrast to Golden Gate and derived methods, overlap-depended assembly methods, such as circular polymerase extension cloning (CPEC) [28], uracil-specific excision reagent cloning (USER) [29], Gibson assembly [30], NeBuilder HiFi assembly (NEB), sequence and ligation independent cloning (SLIC) [31], transformation-associated recombination (TAR) cloning [32] and seamless ligation cloning extract (SLiCE) [33], are sequence independent and therefore convenient and efficient when performing multigene cloning (see review [34]). Moreover, they do not require premade standardised biological parts and can thus be used to easily clone and characterize novel functional parts. Therefore, the overlap-based assembly methods and Gibson assembly, in particular, are the most accepted methods within the synthetic biology community [35]. However, so far they have not been widely adopted in plant sciences, most probably due to the lack of software tools to facilitate the design of complex constructs as well as the lack of cloning parts and vectors, depended on highly efficient overlap-based cloning methods, which would build the basis for the assembly and delivery of multiple-gene cassettes into plants cells.

Recently, Hochrein et al. (2017) [36] developed the AssemblX toolkit, a new cloning strategy supported by the AssemblX web tool. AssemblX enables the assembly of subunits into a multigene structure. The predefined parts order is based on overlaps between adjacent assembly units. Specific overhang sequences first need to be introduced into subunits (e.g. promoter, CDS, terminator) by PCR using custom-designed primers. These primers contain overlaps to the neighbouring fragment in their 5’ region to allow the assembly into entry Level 0 vectors by using overlap-dependent cloning methods. The so-assembled gene cassettes can be combined into the multigene structure in a Level 1 AssemblX vector. To facilitate the transfer of assembly units between different assembly Levels without relying on PCR amplification, rare-cutting restriction enzymes are employed. A multitude of Level 0 and Level 1 vectors is available to allow the assembly of currently up to 25 expression cassettes in a user-defined order. Although the AssemblX toolbox is designed to support easy transfer to any organism, the Level 2 AssemblX vectors are currently available only for bacteria (*Escherichia coli*) and yeast (*Saccharomyces cerevisiae)* [36].

Recently, we developed a design strategy for plant expression vectors and we implemented it, as a grammar, in the Computer-Aided Design (CAD) software GenoCAD [37]. This software tool allows the user to quickly design genetic constructs based on the notion of genetic parts, thereby laying a foundation for the set-up of overlap-dependent assembly methods in plants [38]. The grammar includes a library of plant biological parts organized in structural categories and a set of rules describing how to assemble these parts into large constructs thus minimizing the risk of introducing errors [37].

The aim of our work was to develop tools allowing full flexibility and fast and efficient assembly cloning for multiple protein expression in plants and thus contribute to filling the knowledge gap that impedes the burst of plant synthetic biology. We here present Plant X-tender, an extension of the AssemblX system for the assembly and expression of multigene constructs in plants. Plant X-tender consists of a set of plant expression vectors. We additionally developed tools to support researchers in plant synthetic biology with the extended GenoCAD plant grammar and protocols for most efficient cloning into the novel vector set. The Plant X-tender enables transfer of multigene constructs from AssemblX vectors to plant expression vectors and delivery of multiple-gene cassettes into plant cells in an easy and scalable manner. Our proof of principle experiments pave the way for more complex and increasingly flexible approaches for large-scale engineering in plant synthetic biology.

## Materials and methods

### *In vitro* DNA assembly cloning reactions

SLiCE extract was prepared as described elsewhere [39] with some modifications. Cells were grown in 100 ml 2 x YT medium in 250 ml baffled flasks to OD_600_ = 2. SLiCE reactions were performed as described by Zhang (2012) [32] with 50–500 ng of linear vector and an appropriate amount of insert DNA ranging from 1: 2 to 1: 20 vector to insert molar ratio.

Assembly with NEBuilder HiFi assembly master mix or Gibson assembly master mix was performed according to manufacturer’s recommendations (NEB). Assembly reaction contained 50–500 ng of linear vector, an appropriate amount of insert DNA in a 1: 2 to 1: 10 vector to insert molar ratio and 2x NEBuilder HiFi or Gibson DNA Assembly Master Mix. Assembly reactions were incubated at 50°C for 1h.

Appropriate volumes of the SLiCE, NEBuilder HiFi or Gibson DNA assembled products were transformed into TOP10 *E*. *coli* (Invitrogen) or NEB5α (NEB) by electroporation or heat shock according to the manufacturer’s protocols. Transformed cells were spread on plates containing appropriate antibiotic, ampicillin (100 μg/ml), spectinomycin (75 μg/ml), kanamycin (50 μg/ml) or rifampicin (20 μg/ml).

### *In vivo* DNA assembly cloning reactions in *S*. *cerevisiae* (TAR)

*In vivo* DNA assembly cloning was performed as described by Hochrein et al. (2017) [36]. *Saccharomyces cerevisiae* transformation was performed according to the LiAc/SS carrier DNA/PEG method [40] using strains YPH500 (ATCC^®^ 76626^™^) or BY4741 (ATCC^®^ 201388^™^). The plasmids were isolated from positive colonies using Zymoprep Yeast Miniprep II kit (Zymo Research Corporation) and transformed into electrocompetent ElectroMAX^™^ DH5α-E^™^ *E*. *coli* (Thermo Fisher Scientific).

### Design of AssemblX constructs for plant expression

When needed to be assembled from different subunits, the expression cassettes for Level 0 were designed *in silico* using the extended plant grammar implemented in GenoCAD (http://www.genocad.com/). Sequences were exported as GenBank format and imported into AssemblX web tool (www.assemblx.org) for the design and virtual assembly of multigene constructs and the primer design.

### Construction of Plant X-tender expression vectors

**Fig 1:**
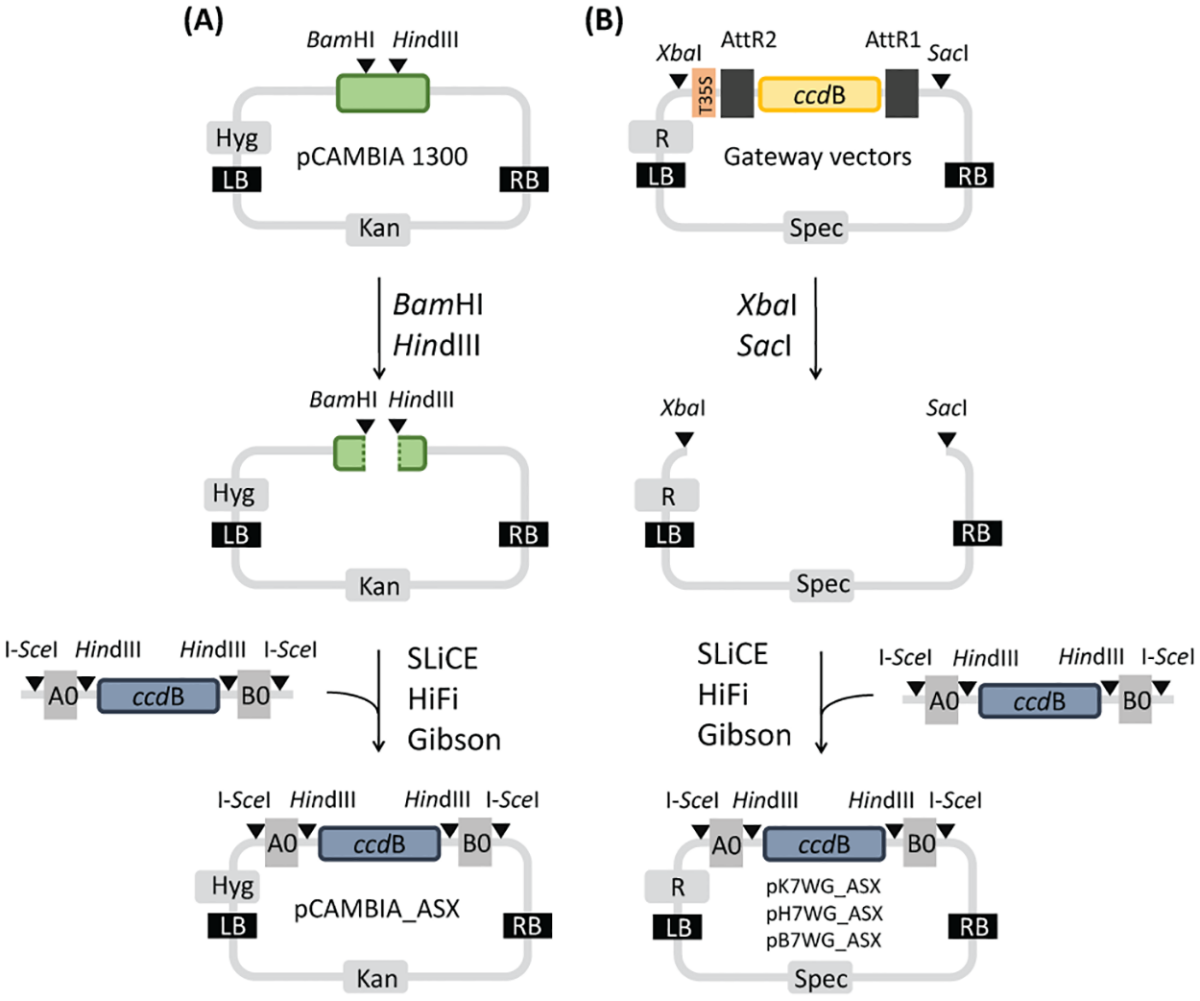
Design of Plant X-tender expression vectors. Vector pCAMBIA 1300 (A) or Gateway vectors (pK7WG, pH7WG or pB7WG) (B) were used as a backbone. (A) I-*Sce*I–A0–*Hin*dIII–*ccd*B–*Hin*dIII–B0–I-*Sce*I cassette was introduced into the MCS region of pCAMBIA1300 by overlap-based cloning methods after backbone digestion with *Bam*HI and *Hin*dIII to obtain pCAMBIA_ASX. (B) T35S–AttR2–*ccd*B–AttR1 cassette was released from the Gateway plasmid backbone by digestion with *Xba*I and *Sac*I and replaced with a I-*Sce*I–A0–*Hin*dIII–*ccd*B–*Hin*dIII–B0–I-*Sce*I cassette by overlap-based cloning methods to obtain pK7WG_ASX, pH7WG_ASX or pB7WG_ASX. MCS: multiple cloning site, A0/B0: homology regions, Kan: selection marker conferring kanamycin resistance in *E*. *coli* and *A*. *tumefaciens*, Spec: selection marker conferring spectinomycin resistance in *E*. *coli* and *A*. *tumefaciens*, Hyg: selection marker conferring hygromycin resistance in plants, R: selection marker conferring resistance in plants (kanamycin resistance in pK7WG, hygromycin resistance in pH7WG, herbicide glufosinate-ammonium resistance in pB7WG), LB: left border of T-DNA, RB: right border of T-DNA, *ccd*B: bacterial suicide gene, *Hin*dIII, I-*Sce*I, *Bam*HI, *Xba*I, *Sac*I: restriction enzyme recognition sites, AttR1/AttR2: Gateway cloning recombination sites, T35S: cauliflower mosaic virus CaMV 35S terminator, SLiCE: Seamless ligation cloning extract cloning method, HiFi: NEBuilder HiFi DNA assembly method, Gibson: Gibson DNA assembly method.

Vector pCAMBIA1300 (Marker Gene Technologies, M1591) was digested with *Bam*HI and *Hin*dIII (NEB) and purified from the gel (Fig 1A). Vectors pK7WG, pB7WG and pH7WG (Karimi et al., 2002) were digested with *Xba*I and *Sac*I (NEB), allowing the release of T35S–AttR2–*ccd*B–AttR1 cassette from the vector backbone and purified from the gel (Fig 1B). The *ccd*B region was amplified from AssemblX pL0A_0–1 Level 0 plasmid [36] using KG15/KG16 primers, purified from the gel and amplified with KG15/KG18 for pCAMBIA1300 and KG19/KG21 primers for the other three plasmid backbones to add homology regions with flanking restriction enzyme recognition sites (see S1 Table for the list of primers). The gel-isolated I-*Sce*I–A0–*Hin*dIII–*ccd*B–*Hin*dIII–B0–I-*Sce*I cassette was assembled into the purified plasmid backbones using different assembly methods and transformed into commercial or homemade One Shot^®^ *ccd*B Survival^™^ 2 T1^R^ Competent Cells (Thermo Fisher Scientific). Transformation efficiencies of homemade competent *E*. *coli* are listed in S2 Table. We confirmed the functionality of *ccd*B gene by transformation of *ccd*B Survival *E*. *coli* strain which allowed propagation of plasmids containing the *ccd*B gene. Transformation of *ccd*B-sensitive *E*. *coli* strain which precludes propagation of plasmids containing the *ccd*B gene with purified plasmids was performed as a control. We verified the correct constructions of the Plant X-tender expression vectors by sequencing the homology regions A0 and B0, I-*Sce*I and *Hin*dIII recognition sites and the *ccd*B gene. We deposited the nucleotide sequences of constructed vectors pCAMBIA_ASX, pK7WG_ASX, pH7WG_ASX and pB7WG_ASX in GenBank under accession numbers MG561370-MG561373. The Plant X-tender expression vectors and their maps are available from Addgene (Addgene IDs 98888-98891).

### Construction of assembly cassettes and cloning into Plant X-tender expression vectors

pL0A_0–1, pL0A_0-R and pL0A_1-R Level 0 vectors [36] were linearized with *Hin*dIII as described in [36]. Inserts were amplified from templates using Phusion^®^ High-Fidelity DNA Polymerase (NEB) and primers with flanking homologous sequences (S3 Table) according to manufacturer’s protocol. Following agarose gel electrophoresis, inserts were purified from the gel using NucleoSpin Gel and PCR Clean-up (Macherey-Nagel) kits, assembled into Level 0 vectors using NEBuilder HiFi assembly master mix (NEB) and transformed into *E*. *coli* NEB 5α (NEB).

pL1A-hc / pL1A-lc (A0/AR) Level 1 vectors [36] were linearized with *Pac*I (NEB). Expression cassettes were released from Level 0 vectors using *Pme*I (NEB), separated by agarose gel electrophoresis and purified from the gel as described above. Multiple cassettes were assembled into Level 1 vectors using TAR or NEBuilder HiFi. Plasmids were isolated from positive yeast colonies and transformed into electrocompetent ElectroMAX^™^DH5α-E^™^ Cells (Thermo Fisher Scientific). Multigene constructs were released from Level 1 vector with I-*Sce*I (NEB). For inserts with similar length to the backbone, the plasmid was additionally digested with *Nhe*I (NEB) to allow separation of the insert and the backbone by gel electrophoresis. Following isolation from the gel using Wizard^®^ SV Gel and PCR Clean-Up System (Promega), inserts were assembled into *Hin*dIII linearized Plant X-tender expression vectors by NEBuilder HiFi or SLiCE assembly method. Constructs were transformed into One Shot^®^ TOP10 Chemically Competent *E*. *coli* (Thermo Fischer Scientific), homemade TOP10 chemically competent *E*. *coli* or homemade TOP10 electrocompetent *E*. *coli*. Incorrect assemblies were selected against by the expression of a suicide gene and by antibiotic selection.

### Colony PCR and sequencing

Positive assemblies were confirmed by colony PCR using KAPA Taq PCR Kits (Kapa Biosystems) or Maxima Hot Start Green PCR Master Mix (Thermo Fisher Scientific) following the manufacturer’s protocols. For further verification, plasmids were isolated from positive colonies using GeneElute Plasmid Miniprep Kit (Sigma) or NucleoSpin Plasmid Easy Pure (Macherey-Nagel) and analysed by sequencing. DNA concentration and purity were evaluated using NanoDrop ND1000 spectrophotometer (Nanodrop technologies). Plasmid DNA and oligonucleotides were prepared according to service provider requirements (LGC Genomics or GATC service provider). Sequences were analysed with CLC Main Workbench (Qiagen). Primers used for colony PCR and sequencing are listed in S4 and S5 Tables.

### *Nicotiana benthamiana* transient transformation

*N*. *benthamiana* seeds (obtained from prof. Van der Vlugt, Wageningen University and Research Centre) were soaked in gibberellic acid overnight to induce germination. Next day the seeds were rinsed three times with sterile water, transferred to soil and grown under controlled environmental conditions as previously described [41]. Five weeks old plants were used for transient transformation. Constructs were introduced into homemade electrocompetent *Agrobacterium tumefaciens* GV3101 by electroporation (Eppendorf Electroporator 2510) following manufacturer’s procedure at 2000 V and confirmed by colony PCR. The transformed cells were cultured to an OD_600_ = 0.5, harvested by centrifugation, resuspended in 0.2 mM acetosyringone water solution (prepared from 200 mM acetosyringone in DMSO) to OD_600_ = 0.5 and mixed with *A*. *tumefaciens* transformed with silencing suppressor p19 (kindly provided by prof. Jacek Hennig) in the ratio 1: 1. The mixture was infiltrated into the second fully developed bottom leaf of *N*. *benthamiana* plants. Empty *A*. *tumefaciens* GV3101 was used as a control.

### Confocal microscopy

Expression of fluorescent proteins was followed six days after agroinfiltration using Leica TCS SP5 laser scanning confocal microscope mounted on a Leica DMI 6000 CS inverted microscope (Leica Microsystems) with an HC PL FLUOTAR 10x objective with zoom factor 1 or 3.05. The 405 nm and 543 nm laser lines were used for the excitation of the enhanced cyan fluorescent protein (ECFP) and monomeric red fluorescent protein 1 (mRFP1). The ECFP emission was measured in the window from 450 to 530, while the mRFP1 was measured in the window from 570 to 630 nm. Three regions of interest per one agroinfiltrated area were scanned bidirectionally with a resolution of 512 x 512 pixels, line average 3 and scan speed 400 Hz. Image merging of brightfield with maximum projections from Z-stacks was performed using Leica LAS AF Lite software (Leica Microsystems).

## Results

### The Plant X-tender toolbox for the assembly of multigene constructs

The AssemblX toolkit contains vectors of three different levels and an accompanying online tool [36]. We expanded the toolkit by developing the Plant X-tender toolbox. The toolbox consists of four newly constructed Plant X-tender plant expression vectors. To facilitate its use in synthetic biology approaches in plant science, the developed vector series is complemented by the *in silico* design tool GenoCAD and protocols for most efficient cloning into the novel vector set.

**Fig 2:**
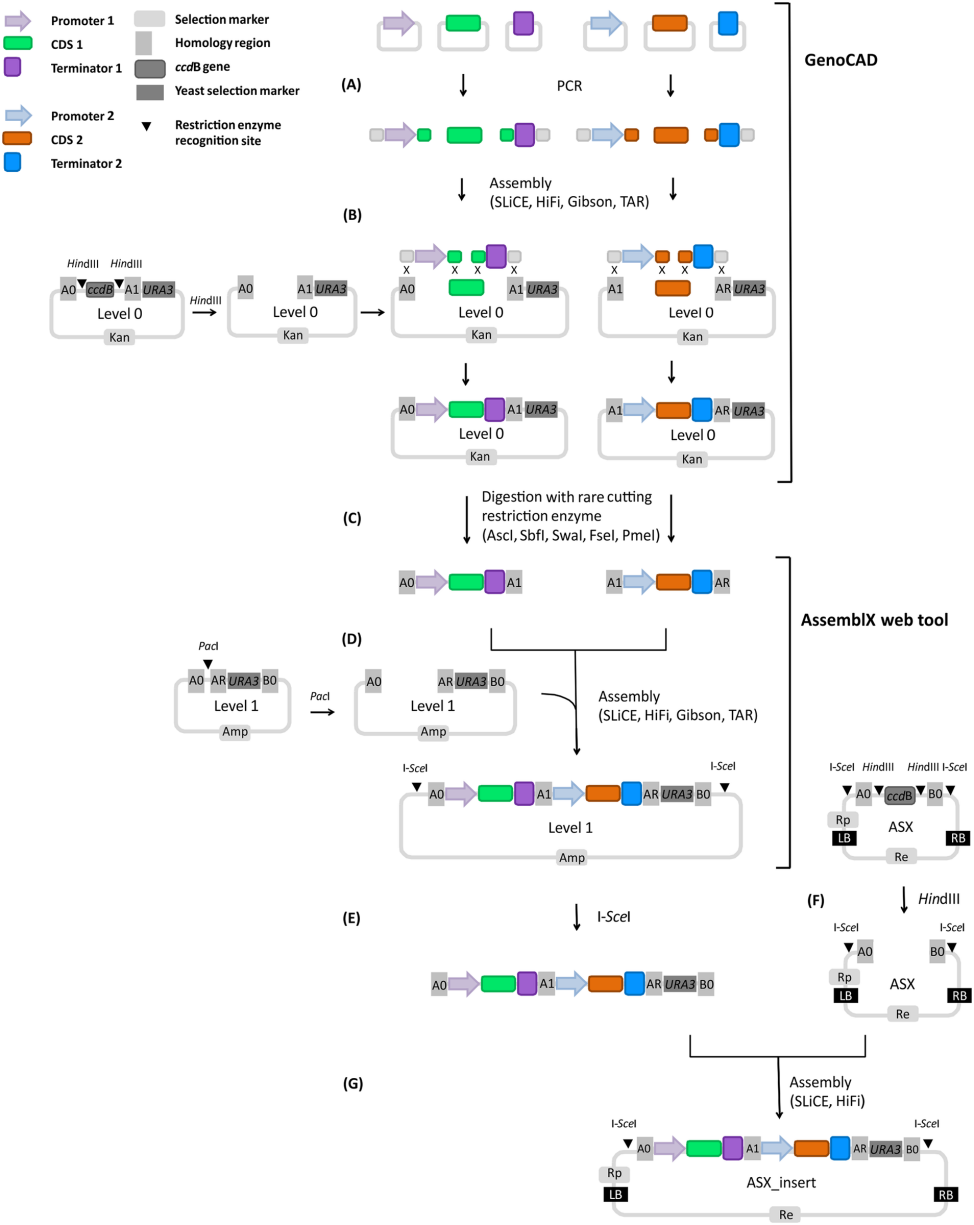
Plant X-tender cloning strategy. Diagram showing example of assembly of two expression cassettes into a plant expression vector using Plant X-tender. Definition of parts and design of Level 0 units is done using GenoCAD. Design of multigene cassettes and computation of primers is performed using the AssemblX webtool. (A-D) Assembly of two expression cassettes into a Level 1 vector. (A) PCR amplification of subunits (e.g. promoter, CDS, terminator) using custom-designed primers with appropriate 5’ extensions to add overlaps between the individual subunits and chosen Level 0 plasmid. (B) Assembly of subunits into *Hin*dIII digested Level 0 vectors via overlap-based assembly methods. Only the restriction of Level 0 vector with A0/A1 homology regions is shown. (C) Assembled cassettes flanked by homology regions are released from the backbone using one of five rare 8-base cutter recognition sites (*Asc*I, *Sbf*I, *Swa*I, *Fsa*I, *Pme*I) flanking the homology regions. (D) Assembly of expression cassettes into *Pac*I digested Level 1 vector by of the preferred overlap-based assembly method. (E-G) Multigene assembly into Plant X-tender expression vector. (E) Digestion with I-*Sce*I allows the release of a multigene construct flanked by homology regions A0 and B0 from the Level 1 AssemblX vector. (F) *Hin*dIII digestion enables the linearization of Plant X-tender expression vector and the release of *ccd*B cassette prior the assembly. (G) Assembly of a multigene construct and a yeast selection marker (*URA3*) flanked by homology regions into Plant X-tender expression vector by overlap-based methods exploiting homologous recombination between the homology regions A0 and B0 of the Plant X-tender expression vector and the homology regions A0 and B0 of the insert. A0, A1, AR, B0: homology regions, *Hin*dIII, I-*Sce*I, *Pac*I, *Asc*I, *Sbf*I, *Swa*I, *Fse*I, *Pme*I: restriction enzyme recognition sites, Rp: selection marker conferring resistance in plants, Re: selection marker conferring resistance in *E*. *coli* and *A*. *tumefaciens*, Amp: selection marker conferring ampicillin resistance in *E*. *coli* and *A*. *tumefaciens*, Kan: selection marker conferring kanamycin resistance in *E*. *coli* and *A*. *tumefaciens*, *URA3*: yeast selection marker, LB: left border of T-DNA, RB: right border of T-DNA, *ccd*B: bacterial suicide gene, SLiCE: Seamless ligation cloning extract cloning method, HiFi: HiFi DNA assembly method, Gibson: Gibson DNA assembly method, TAR: cloning based on transformation-associated recombination, PCR: Polymerase chain reaction, CDS: coding sequence, ASX: Plant X-tender expression vector.

We first customized the plant grammar implemented in GenoCAD [37] by adding sequences of nopaline synthase promoter (pNOS) and cauliflower mosaic virus CaMV 35S terminator (t35S) to the GenoCAD plant library. We constructed an additional plant grammar rule to facilitate virtual assembly of the selected biological parts into the expression cassette. The new rule allows the design of a simple expression cassette segment without the whole vector. The user is guided through the design of an expression cassette that, as a minimum requirement, includes promoter, CDS and terminator. However, it offers flexibility with regard to the CDS, which can represent a plant gene, a fluorescent protein or both, and can be fused to epitope tags and linkers, if needed. The user can also decide to add one or two promoters and terminators. Moreover, it includes a library of plant genetic parts classified in structural categories which constitute a repository of biological parts sharable with the plant community and easily accessible for all the users. The newly developed grammar is available at Figshare (https://doi.org/10.6084/m9.figshare.4977464) and can be imported into the GenoCAD. We used newly developed grammar to virtual assemble the selected biological parts into the expression cassette (Fig 2, GenoCAD). Thus, the GenoCAD plant grammar complements the AssemblX web tool by supporting the user throughout the design of Level 0 constructs. In the next step, we exported the sequences of the designed expression cassettes from GenoCAD and imported them into the AssemblX web tool to virtually assemble the expression cassettes in Level 1 AssemblX vectors in order to combine them in a multigene construct (Fig 2, AssemblX web tool). Finally, we generated the plasmid maps of the intended final product by cutting and pasting the assembly module from Level 1, flanked by A0 –B0 homology regions, into the final destination plant expression vector. In general, specific overlapping homology regions (A0 and B0 in this study) are incorporated into the assembly module by restriction digestion of Level 1 AssemblX vector using rare-cutting restriction enzyme. Introduced homology regions will later allow the scar-less assembly into Plant X-tender expression vector containing the same homology regions.

The Plant X-tender cloning procedure follows the AssemblX strategy (Fig 2). The strategy relies on overlap-based cloning methods which utilize various overlapping homology regions flanking the DNA parts to allow the scar-less assembly of multiple parts into a single DNA construct. Specific overhang sequences first need to be introduced into subunits (e.g. promoter, CDS, terminator) by PCR using custom-designed primers overlapping the neighbouring fragment in their 5’ region to allow assembly based on overlap-dependent cloning methods and to determine the orientation and order of DNA fragments in AssemblX Level 0 vectors (Fig 2A and 2B). Level 0 vectors differ in two variable homology regions that will later define the position of the assembly unit within the final construct. The so-assembled gene cassettes are combined into a multigene structure in a Level 1 AssemblX vector (Fig 2C and 2D). Different Level 1 vectors are compatible with different Level 0 vector sets depending on the homology regions they contain. In the last step, the construct is transferred into newly developed Plant X-tender expression vectors exploiting homologous recombination between the homology regions of the expression vector and the homology regions of the construct, thus allowing introduction of up to five expression cassettes into a single plasmid (Fig 2E–2G). Although it is highly recommended to use the plant grammar implemented in GenoCAD and AssemblX web tool for the design to decrease the risk of introducing errors, one or both design tools could be omitted to apply cut-and-paste approach.

### Validating the Plant X-tender expression vectors

**Fig 3:**
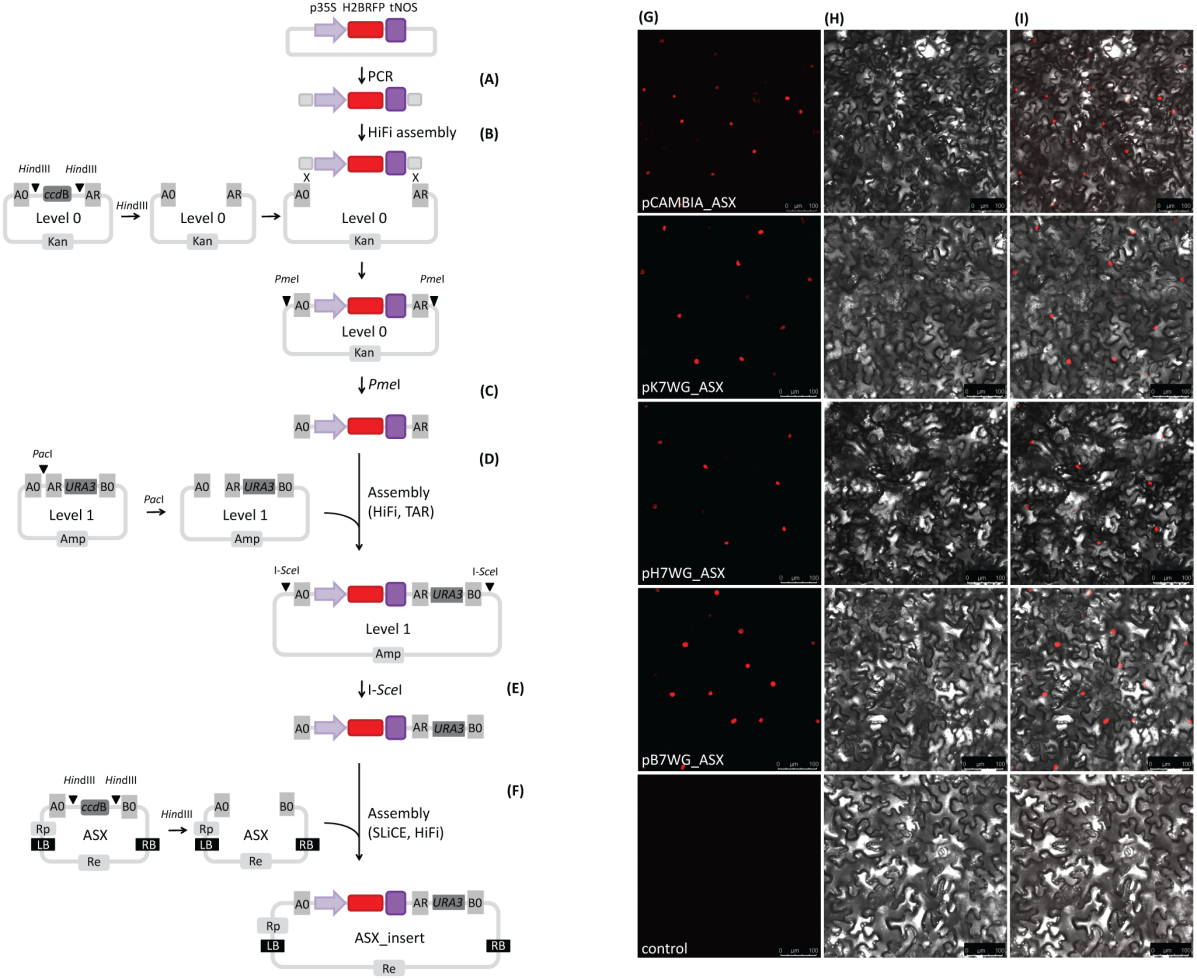
Functional evaluation of constructed vectors by cloning expression cassette p35S::H2BRFP_tNOS into Plant X-tender expression vectors. (A-F) Scheme of the cloning procedure. (A) Amplification of expression cassette from template plasmid using primers with appropriate 5’ and 3’ extensions to add A0 and AR homology regions. (B) Expression cassette assembly in *Hin*dIII restricted pL0A_0-R Level 0 vector by NEBuilder HiFi assembly method. (C) Release of expression cassette with flanking homology regions A0 and AR from Level 0 vector by *Pme*I digestion. (D) Assembly of expression cassette with flanking homology regions A0 and AR into *Pac*I digested pL1A-hc / pL1A-lc (A0/AR) Level 1 vector by TAR or NEBuilder HiFi. (E) Release of expression cassette flanked by *URA3* yeast selection marker and homology regions A0 and B0 from Level 1 vector by I-*Sce*I digestion. (F) Assembly of expression cassette flanked by *URA3* yeast selection marker and homology regions A0 and B0 into Plant X-tender expression vectors by SLiCE or NEBuilder HiFi. (G-I) Images of agroinfiltrated *N*. *benthamiana* leaves obtained by laser scanning confocal microscopy. Leaves were agroinfiltrated with agrobacteria containing pCAMBIA_ASX_cassette, pK7WG_ASX_cassette, pH7WG_ASX_cassette, pB7WG_ASX_cassette or empty agrobacteria (top to bottom). (G) Nuclear localisation of RFP. Fluorescence is represented as maximum projections of z-stacks. (H) Bright field. (I) Overlay of G with H. Scale bars are 100 μm. p35S: cauliflower mosaic virus CaMV 35S promoter, H2BRFP: histon sequence fused to red fluorescence protein (mRFP1), tNOS: nopaline synthase terminator, A0, AR, B0: homology regions, Rp: selection marker conferring resistance in plants (hygromycin in the case of pCAMBIA_ASX and pH7WG_ASX, kanamycin in the case of pK7WG_ASX, glufosinate-ammonium in the case of pB7WG_ASX), Re: selection marker conferring resistance in *E*. *coli* and *A*. *tumefaciens* (kanamycin in the case of pCAMBIA_ASX, spectinomycinin in the case of pK7WG_ASX, pH7WG_ASX and pB7WG_ASX), Amp: selection marker conferring ampicillin resistance in *E*. *coli* and *A*. *tumefaciens*, Kan: selection marker conferring kanamycin resistance in *E*. *coli* and *A*. *tumefaciens*, LB: left border of T-DNA, RB: right border of T-DNA, *Hin*dIII, I-*Sce*I, *Pac*I, *Pme*I: restriction enzyme recognition sites, *URA3*: yeast selection marker, *ccd*B: bacterial suicide gene, SLiCE: Seamless ligation cloning extract cloning method, HiFi: NEBuilder HiFi DNA assembly method, Gibson: Gibson DNA assembly method, TAR: cloning based on transformation-associated recombination, PCR: Polymerase chain reaction, ASX: Plant X-tender expression vector.

We tested the developed Plant X-tender toolbox by cloning the expression cassette p35S::H2BRFP_tNOS into newly developed Plant X-tender expression vectors. We further confirmed the functionality of the system by transient expression of H2BRFP in *N*. *benthamiana* (Fig 3). This expression cassette, which consists of a histon sequence fused to red fluorescent protein mRFP1, was already confirmed to be functional [42], therefore we used it as the proof of concept. We amplified the expression cassette from a template plasmid using primers with appropriate 5’ and 3’ extensions, giving homology to A0 and AR homology regions and assembled it in pL0A_0-R Level 0 vector [36] by NEBuilder HiFi assembly (Fig 3A and 3B). We confirmed that eight out of eight colonies contained the plasmid with DNA insert by colony PCR. Additionally, the correct junction sites in NEBuilder HiFi assembled plasmid were confirmed by sequencing. Subsequently, following the AssemblX procedure, we assembled the resulting Level 0 unit into pL1A-hc / pL1A-lc (A0/AR) Level 1 vector [36] by TAR and NEBuilder HiFi assembly to compare the efficiency of both (Fig 3C and 3D). For TAR, all clones analysed by colony PCR were confirmed to contain the correct insert length, while NEBuilder HiFi assembly resulted in less than 60% of clones with the correct insert length. The correct junction sites of TAR assembled plasmid were confirmed by sequencing. In the next step, we tested several cloning and transformation parameters for transferring the assembled expression cassette (Level 1 module) from the AssemblX Level 1 vector to the Plant X-tender expression vector pCAMBIA_ASX (Fig 3E and 3F). Transfer parameters tested were the cloning method, amount of the plasmid backbone, the molar ratio between the plasmid backbone and the insert, transformation method, *E*. *coli* strain, transformation efficiency of *E*. *coli* and the volume of cloning mixture used for transformation (S6 Table). Our results proved SLiCE assembly to be more efficient compared to NEBuilder HiFi assembly when 50 ng of the plasmid and a 1: 2 molar ratio between the plasmid and the insert were used. We obtained a higher number of colonies when the higher amount of the plasmid was used (150 ng in comparison to 50 ng). Likewise, a higher molar ratio of insert to the plasmid (10: 1 in comparison to 2: 1) resulted in higher transformation efficiency. Our results showed that SLiCE cloning method is applicable even in combination with homemade chemically competent or electrocompetent *E*. *coli* with lower transformation efficiency in comparison to expensive commercial ones (S2 and S6 Tables). We further transferred the expression cassette p35S::H2BRFP_tNOS into pK7WG_ASX, pH7WG_ASX and pB7WG_ASX vectors using the most favourable assembly method determined by the pCAMBIA_ASX transfer, SLiCE (Fig 3C–3F). We confirmed the functionality of the constructs by the confocal imaging of H2BRFP upon agroinfiltration of *N*. *benthamiana* leaves (Fig 3G–3I).

### Efficient multigene cassettes cloning with Plant X-tender

**Fig 4:**
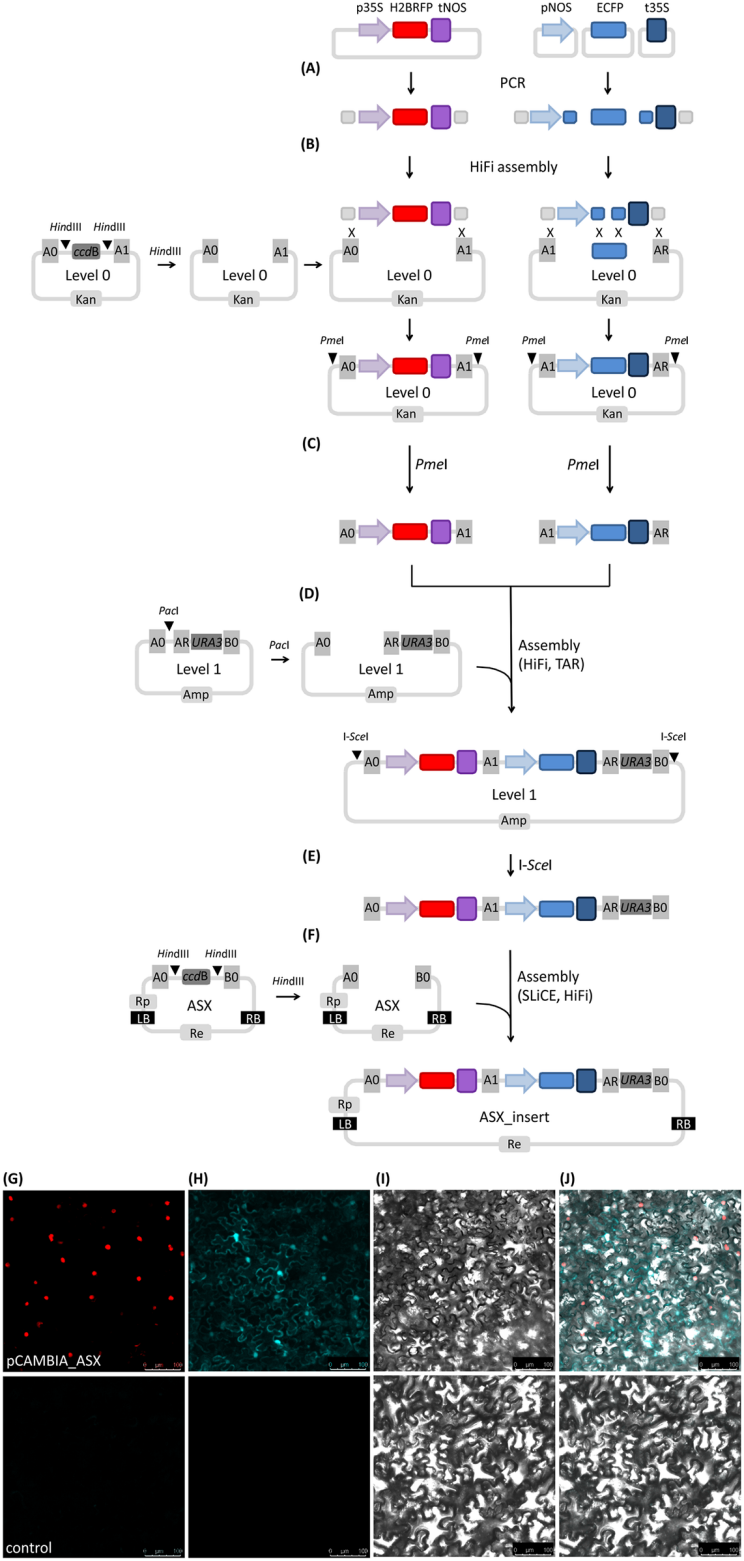
Multigene cloning with Plant X-tender expression vectors. Two expression cassettes were cloned into pCAMBIA_ASX and introduced into *N*. *benthamiana*. (A-F) Scheme of cloning procedure. (A) Amplification of expression cassette from template plasmid using primers with appropriate 5’ and 3’ extension homologies in the case of p35S::H2BRFP_tNOS expression cassette. PCR amplification of subunits (pNOS, ECFP, t35S) using custom-designed primers with appropriate 5’ extensions to add overlaps between the individual subunits and chosen Level 0 plasmid in the case of pNOS::ECFP_t35S expression cassette. (B) Assembly of subunits into *Hin*dIII digested Level 0 vectors by NEBuilder HiFi assembly method. Only the restriction of Level 0 vector with A0/A1 homology regions is shown. (C) Assembled cassettes flanked by homology regions were released from the backbone using *Pme*I. (D) Assembly of expression cassettes into *Pac*I digested Level 1 vector by TAR or NEBuilder HiFi. (E) Release of the multigene construct from Level 1 vector using I-*Sce*I homing endonuclease, cutting outside the homology regions A0 and B0. (F) Assembly of two expression cassettes and yeast selection marker (*URA3*) into *Hin*dIII digested Plant X-tender expression vectors with SLiCE of NEBuilder HiFi. (G–J) Images of agroinfiltrated *N*. *benthamiana* leaves obtained by laser scanning confocal microscopy. Leaves were agroinfiltrated with agrobacteria containing pCAMBIA_ASX_multigene (upper panel) or with empty *A*. *tumefaciens* (bottom panel). (G) Nuclear localisation of RFP. Fluorescence is represented as a maximum projection of z-stacks. (H) ECFP is localised in the cytoplasm. Fluorescence is represented as maximum projections of z-stacks. (I) Bright field. (J) Overlay of G, H and I. Scale bars are 100 μm. p35S: cauliflower mosaic virus CaMV 35S promoter, H2BRFP: histon sequence fused to red fluorescence protein (mRFP1), tNOS: nopaline synthase terminator, pNOS: nopaline synthase promoter, ECFP: cyan fluorescent protein, t35S: cauliflower mosaic virus CaMV 35S terminator, A0, A1 AR, B0: homology regions, Rp: selection marker conferring hygromycin resistance in plants, Re: selection marker conferring kanamycin resistance in *E*. *coli* and *A*. *tumefaciens*, Amp: selection marker conferring ampicillin resistance in *E*. *coli* and *A*. *tumefaciens*, Kan: selection marker conferring kanamycin resistance in *E*. *coli* and *A*. *tumefaciens*, LB: left border of T-DNA, RB: right border of T-DNA, *Hin*dIII, I-*Sce*I, *Pac*I, *Asc*I, *Sbf*I, *Swa*I, *Fse*I, *Pme*I: restriction enzyme recognition sites, *URA3*: yeast selection marker, *ccd*B: bacterial suicide gene, SLiCE: Seamless ligation cloning extract cloning method, HiFi: NEBuilder HiFi DNA assembly method, Gibson: Gibson DNA assembly method. TAR: cloning based on transformation-associated recombination, PCR: Polymerase chain reaction, ASX: Plant X-tender expression vector.

Finally, we tested the usability of Plant X-tender for multigene cloning by introducing two expression cassettes, containing two different reporter genes (i.e. H2BRFP and ECFP), into newly developed Plant X-tender expression vectors. We confirmed the functionality of the system by transient expression of H2BRFP and ECFP in *N*. *benthamiana* (Fig 4). We amplified the p35S::H2BRFP_tNOS expression cassette from the template using primers with appropriate 5’ and 3’ extensions to add A0 and A1 homology regions and assembled it in Level 0 vector pL0A_0–1 [36] by NEBuilder HiFi assembly. In the case of the pNOS::ECFP_t35S expression cassette, we amplified each Level 0 subunit from separate templates using primers with overlaps between adjacent subunits and destination plasmid. We assembled the three subunits in Level 0 vector pL0A_1-R [36] by NEBuilder HiFi assembly (Fig 4A and 4B). We used GenoCAD and the AssemblX webtool to design the construct and the cloning primers. We confirmed that eight out of eight colonies contained the insert for both Level 0 assemblies by colony PCR. The correct junction sites in the assembled plasmids were confirmed by sequencing. We subsequently assembled both expression cassettes in Level 1 vector pL1A-hc / pL1A-lc (A0/AR) [36] by TAR and NEBuilder HiFi assembly (Fig 4C and 4D). According to the colony PCR results, TAR assembly was confirmed to be more efficient in comparison to NEBuilder HiFi assembly in the conditions tested here. For TAR assembly, all clones were confirmed to contain the correct insert length, while NEBuilder HiFi assembly resulted in less than 70% of clones with the correct insert length in the conditions tested here. The multigene construct consisting of both expression cassettes with flanking homology regions A0/B0 was released from Level 1 vector and cloned into pCAMBIA_ASX vector (Fig 4E and 4F). We evaluated different cloning and transformation methods for this step. The lower molar ratio of insert to plasmid was determined to be more efficient. In the tested conditions, NEBuilder HiFi cloning was determined as not applicable in combination with electroporation, while SLiCE could be used in combination with electroporation or heat shock. SLiCE cloning was confirmed to be applicable even in combination with low efficient homemade chemically competent *E*. *coli* cells (S6 Table). We confirmed the functionality of the construct by confocal imaging of H2BRFP for the p35S::H2BRFP_tNOS expression cassette and ECFP for the pNOS::ECFP_t35S expression cassette (Fig 4G–4J), upon agroinfiltration of *N*. *benthamiana* leaves.

## Discussion

There is a growing need for simple but flexible cloning strategies, which will allow high throughput approaches in plant synthetic biology [1] and biotechnology [43]. We developed Plant X-tender, a set of tools that complements the AssemblX toolkit [36] and thus enables easier development of plant synthetic biology applications. The Plant X-tender integrates and expands already existing tools, instead of providing a new but isolated cloning procedure. This is especially beneficial for researchers working with different organisms in parallel, as it will enable the use of a single cloning scheme for different organisms, including plants.

When designing Plant X-tender expression vectors, attention was focused on providing full flexibility regarding the design of the expression cassettes. The selection of appropriate regulatory elements is crucial since the repetitive use of the same promoter for expression of multiple genes was shown to be associated with transgene silencing [44,45,46]. In addition, in the AssemblX system, the cloning steps for each assembly reaction are designed in a user-oriented workflow, allowing the user to freely choose the final orientation of modules and thus avoid gene silencing driven by inverted repeats [21,44,47,48]. Moreover, this planning strategy allows the use of linkers between expression cassettes in order to improve expression, folding and/or stability of the proteins when expressing recombinant fusion proteins [49]. Although a few plant expression plasmids for multigene cloning have already been developed, they are rarely fully flexible regarding regulatory elements, orientation of expression cassettes and addition of linkers between them, if required. With these problems in mind, Plant X-tender expression vectors are available as empty backbones.

Another advantage of the Plant X-tender vector system presented here is the flexibility in the choice of a selection marker. Since there are reports on interactions between selection markers and gelling agents [50] as well as connections between selection markers and regenerability [51], our set of Plant X-tender expression vectors, including three expression vectors with the same backbone (pK7WG_ASX, pH7WG_ASX and pB7WG_ASX) but different selection markers, enables the selection of the most appropriate selection marker matching the target tissue as well as transformation media. According to our experience, the backbone can affect the expression pattern as well as transformation efficiency. Therefore, we constructed an additional Plant X-tender expression vector, pCAMBIA_ASX, with the intention of providing flexibility in terms of backbones.

One major advantage of the novel vector set is the inclusion of the *ccd*B gene (Fig 1), which precludes growth of non-recombinant clones [52] and makes the screening easier and more efficient. The other is the ability to assemble scar-free constructs, which is especially important since the introduced sequences can affect transgene function [27] and was also implemented in recently developed COLORFUL-circuit vectors [21].

Although restriction enzyme-based methods derived from the Golden Gate strategy share a number of characteristics that encourage their adoption by the scientific community, they have one disadvantage in common. All parts are required to be devoid of any restriction sites used in the assembly. This could pose a problem, as Ghareeb et al. (2016) [21] noticed a high occurrence of restriction enzyme recognition sites used in Golden Gate technology in several plant genomes. To overcome this downside of restriction enzyme-based methods, Ghareeb et al. (2016) [21] exploited the use of a newly discovered rare-cutting restriction enzyme. Despite the utility and advantages of the system, annealing of short sticky ends may have limited affinity and specificity when assembling multiple DNA parts in one pot (reviewed in [53]). Since the cloning approach presented here is based on overlap-based cloning methods which utilize longer overlapping homology regions the affinity and specificity issue is overcome. Moreover, since our cloning approach is based on rare-cutting restriction enzymes in combination with overlap-based methods, it is sequence independent and thus avoids elimination of unwanted restriction sites. However, the issue of introducing sequence errors and time-consuming parts domestication could nowadays be avoided by DNA synthesis.

To avoid possible confusion, one should note different nomenclature used for the naming of the assembly levels in AssemblX and Type IIS-mediated assembly methods. In AssemblX, the subunits refer to basic elements below the transcriptional unit level (e.g. promoter, CDS, terminator) which are assembled in Level 0 vectors. Therefore, Level 0 refers to single gene cassettes, while Level 1 refers to multigene constructs. On the other hand, Level 0 in MoClo and GoldenBraid [25, 26] usually refers to basic elements, single gene cassettes are Level 1 and multigene constructs are Level 2 assemblies.

Another advantage of the AssemblX-based approach presented here, lies in the flexibility to select the most appropriate method among several overlap-based assembly methods according to the user requirements. To determine the most efficient assembly method and cloning conditions, we evaluated different overlap-based assembly methods for cloning in various conditions. Cloning efficiency of 100% in the case of Level 0 assembly by NEBuilder HiFi for all tested expression cassettes could be attributed to optimized homology regions present in Level 0 vectors and *ccd*B counterselection. In contrast, using TAR assembly method for Level 1 assembly resulted in higher cloning efficiency, if compared to NEBuilder HiFi assembly method in tested conditions and for the selected inserts. Our results are consistent with the results of de Kok et al. (2014) [54], who obtained higher cloning efficiency by TAR assembly in comparison to a highly promoted commercial assembly kit (Gibson assembly). In the last step of the cloning strategy followed here, we assembled the constructs into Plant X-tender expression vectors. Results from the assembly of p35S::H2BRFP_tNOS expression cassette (Fig 2) are in agreement with the results from Zhang et al. (2012) [33], who claimed that increasing the amount of the insert at the same vector amount as well as increasing the amount of the vector and the insert yield higher cloning efficiencies. In contrast, results obtained by the assembly of p35S::H2BRFP_tNOS + pNOS::ECFP_t35S multigene construct (Fig 3) speak in favour of lower molar ratio. The transformation of highly efficient chemically competent *E*. *coli* with a SLiCE assembly mixture containing 150 ng of plasmid and a 2-fold molar excess of insert resulted in higher number of colonies in comparison to the higher molar ratio (1: 5) of vector and insert (S6 Table). Our results from the multigene assembly are consistent with the results from Okegawa and Motohashi (2015) [55] and Motohashi (2015) [56], who observed a significant decrease of cloning efficiency by increasing molar ratio of vector to insert when using SLiCE assembly.

High cloning efficiency could be attributed to optimized homology regions [36] introduced in the Plant X-tender expression vectors and to *ccd*B counterselection. Although SLiCE has originally been used in combination with commercially available highly competent *E*. *coli* cells [33], our results showed that SLiCE is applicable even in combination with low efficient homemade chemically competent or electrocompetent *E*. *coli* (S6 Table), which is in agreement with published data from Motohashi (2015) [56] and Messerschmidt et al. (2016) [39]. However, in the case of more demanding cloning settings or low plasmid concentrations, highly competent *E*. *coli* cells are recommended.

The cloning strategy presented here is designed in three successive cloning levels, allowing multiple parallel assemblies resulting in increasingly complex structure. Although Plant X-tender for AssemblX enables the introduction of large constructs into plant expression vectors, the stability of expression in the plant genome is still highly unpredictable and low. However, as plant genome engineering using synthetic biology is developing fast [57], it is most likely that this problem will be reduced, for example in combination with CRISPR/Cas9 guided insertion of constructs into the plant genome.

Taken together, we here present Plant X-tender that enables easy cloning of multigene cassettes and their expression in plant cells as an extension of the AssemblX toolbox [36]. The system is highly flexible and fast thus allowing easier introduction of synthetic biology into plant science.

## Supporting information

S1 Table Primers used for the construction of Plant X-tender expression vectors. (PDF)

S2 Table Transformation efficiencies of homemade chemically competent and electrocompetent *E*. *coli*. (PDF)

S3 Table Oligonucleotides used for the amplification of Level 0 subunits by PCR. A) Oligonucleotides used for the amplification of Level 0 subunit for the assembly of expression cassette p35S::H2BRFP_tNOS. B) Oligonucleotides used for the amplification of Level 0 subunits for the assembly of multigene construct p35S::H2BRFP_tNOS + pNOS::ECFP_t35S. Nucleotides in bold represent overlaps between adjacent parts, e.g. homology regions of destination plasmid (Level 0 AssemblX vector) or sequence of adjacent modules.
(PDF)

S4 Table List of primers for sequencing and colony PCR. (PDF)

S5 Table Primers for sequencing and colony PCR. (PDF)

S6 Table Optimization of assembly and transformation methods for the insert cloning into Plant X-tender expression vectors. Expression cassette p35S::H2BRFP_tNOS (insert 1) and multigene construct p35S::H2BRFP_tNOS + pNOS::CFP_t35S (insert 2) were separately assembled into pCAMBIA_ASX to determine optimal conditions for insert cloning into the expression vectors. Bottom two rows represent a negative control (the plasmid backbone without the insert). m (ng): amount of pCAMBIA_ASX expression vector, AM: assembly method, SLiCE: Seamless ligation cloning extract cloning method, HiFi: HiFi DNA assembly method, MR: molar ratio between the plasmid backbone and the insert, TM: transformation method, *E*. *coli*: competent *E*. *coli* used for transformation, HM: homemade TOP10 chemically competent *E*.*coli*, HM: homemade TOP10 electrocompetent *E*.*coli*, C: commercial TOP10 chemically competent *E*.*coli*, T (μl): volume of assembly mixture used for transformation, number of colonies: the number of colonies grown after overnight incubation, cloning efficiency: the ratio between the number of clones with the correct insert length confirmed by colony PCR and the number of colonies subjected to colony PCR.
(PDF)
