# Supplementary material for: Plant X-tender: An extension of the AssemblX system for the assembly and expression of multigene constructs in plants
Source: PLoS One. 2018 Jan 4;13(1):e0190526. doi: 10.1371/journal.pone.0190526 (PMC5754074; doi:10.1371/journal.pone.0190526)
Supplement: S1 Table — (PDF) [file pone.0190526.s001.pdf]

S1 Table: Primers used for the construction of Plant X-tender expression vectors.

Primer name	Primer sequence (5' → 3')
KG15_F	CCATGATTACGAATTCGAGCTCGGTACCCGGTAGGGATAACAGGGTAATATCCCTATTGTCCCATTAAAATAGGAAAGTCTGGTAACTCAGG
KG16_R	GGGTAATTGAATAACTGCTCCCGTTGTAAAAGTATCCGTAGTGTGACTGAAACCTTAAAGCTTACTGGCTGTGTATAAGGG
KG18_R	CGTTGTAAAACGACGGCCAGTGCCGTAGGGATAACAGGGTAATTGAATAACTGCTCCCGTTGTAAAAGT
KG19_F	ATAATTCGCGGTACCCGGGGATCCTAGGGATAACAGGGTAATATCCCTATTGTCCCATTAAAATAGGAAAGTCTGGTAACTCAGG
KG21_R	TCTGATCCAAGCTCAAGCTAAGCTGTAGGGATAACAGGGTAATTGAATAACTGCTCCCGTTGTAAAAGT
