# Supplementary material for: Plant X-tender: An extension of the AssemblX system for the assembly and expression of multigene constructs in plants
Source: PLoS One. 2018 Jan 4;13(1):e0190526. doi: 10.1371/journal.pone.0190526 (PMC5754074; doi:10.1371/journal.pone.0190526)
Supplement: S2 Table — (PDF) [file pone.0190526.s002.pdf]

S2 Table: Transformation efficiencies of homemade chemically competent and electrocompetent *E. coli*.

homemade chemically competent and electrocompetent *E. coli*	transformation efficiency
homemade TOP10 electrocompetent *E. coli*	$4{,}22 \times 10^{7}$ cfu/pmol
homemade TOP10 chemically competent *E. coli*	$1{,}87 \times 10^{5}$ cfu/pmol
homemade *ccd*B Survival™ 2 $T1^{R}$ electrocompetent *E. coli*	$1{,}00 \times 10^{8}$ cfu/pmol
homemade *ccd*B Survival™ 2 $T1^{R}$ chemically competent *E. coli*	$4{,}31 \times 10^{6}$ cfu/pmol
