# Supplementary material for: Plant X-tender: An extension of the AssemblX system for the assembly and expression of multigene constructs in plants
Source: PLoS One. 2018 Jan 4;13(1):e0190526. doi: 10.1371/journal.pone.0190526 (PMC5754074; doi:10.1371/journal.pone.0190526)
Supplement: S3 Table — A) Oligonucleotides used for the amplification of Level 0 subunit for the assembly of expression cassette p35S::H2BRFP_tNOS. B) Oligonucleotides used for the amplification of Level 0 subunits for the assembly of multigene construct p35S::H2BRFP_tNOS + pNOS::ECFP_t35S. Nucleotides in bold represent overlaps between adjacent parts, e.g. homology regions of destination plasmid (Level 0 AssemblX vector) or sequence of adjacent modules. (PDF) [file pone.0190526.s003.pdf]

S3 Table: Oligonucleotides used for the amplification of Level 0 subunits by PCR. A) Oligonucleotides used for the amplification of Level 0 subunit for the assembly of expression cassette p35S::H2BRFP_tNOS. B) Oligonucleotides used for the amplification of Level 0 subunits for the assembly of multigene construct p35S::H2BRFP_tNOS + pNOS::ECFP_t35S. Nucleotides in bold represent overlaps between adjacent parts, e.g. homology regions of destination plasmid (Level 0 AssemblX vector) or sequence of adjacent modules.

A

p35S::H2BRFP_tNOS

Primer name	Primer sequence (5' → 3')	Level 0 unit
KG1_F	**CAGGACACTGTATCTGCTACGCTGTTTAT**GATTAGCCTTTTCAATTTCAGAAAGAATGCTAACCC	**A0** p35S::H2BRFP_tNOS
KG2_R	**AAATGATGAATTGAATTGAAAAGCTA**CCCTCACTAAAGGGAACAAAAGCTGG	p35S::H2BRFP_tNOS **AR**

B

Multigene construct / p35S::H2BRFP_tNOS

Primer name	Primer sequence (5' → 3')	Level 0 unit
KG3_F	**GGACACTGTATCTGCTACGCTGTTTAT**GATTAGCCTTTTCAATTTCAGAAAGAATGCTAACCC	**A0** p35S::H2BRFP_tNOS
KG4_R	**GCAGCGTTTATTGTGCGAATCTCACTTTA**CCCTCACTAAAGGGAACAAAAGCTGG	p35S::H2BRFP_tNOS **A1**

Multigene construct / pNOS::ECFP_t35S

Primer name	Primer sequence (5' → 3')	Level 0 subunits
KG5_F	**TTCGCACAATAAACGCTGCTTCTGTT**GAGCGGAGAATTAAGGGAGTCACG	**A1** pNOS
KG6_R	**CCTTGCTCACCAT**GCGAAACGATCCAGATCCGGTGC	pNOS **ECFP**
KG7_F	**TGGATCGTTTCGC**ATGGTGAGCAAGGGCGAGGAGC	**pNOS** ECFP
KG8_R	**TCTAGCATGGCCG**TTACTTGTACAGCTCGTCCATGCCG	ECFP **t35S**
KG9_F	**GCTGTACAAGTAA**CGGCCATGCTAGAGTCCGC	**ECFP** t35S
KG10_R	**AAATGATGAATTGAATTGAAAAGCTA**AGGTCACTGGATTTTGGTTTTAGG	t35S **AR**
