# Supplementary material for: Plant X-tender: An extension of the AssemblX system for the assembly and expression of multigene constructs in plants
Source: PLoS One. 2018 Jan 4;13(1):e0190526. doi: 10.1371/journal.pone.0190526 (PMC5754074; doi:10.1371/journal.pone.0190526)
Supplement: S4 Table — (PDF) [file pone.0190526.s004.pdf]

S4 Table: List of primers for sequencing and colony PCR.

Primer name	Primer sequence (5' → 3')
M13F	GTAAAACGACGGCCAGT
M13R	CAGGAAACAGCTATGAC
PVIBF	TAGGGATAACAGGGTAATATC
PVIBR8527	AACGGCTCTCTCTTTTATAG
FM176	AGCAGCCGATTGTCTGTTGT
FM218	TAGCTCTTGATCCGGCAA
KG022	CACCTTCCTTTTCCACTATCT
FM182	GAAGAAAGCGAAAGGAGC
FM1113	CAAAGACCCCAACGAGAA
KG032	CCTAATGCTTCAACTAACTCC
FM131	AATACGCAAACCGCCTCTC
FM032	GGAGTTAGTTGAAGCATTAGG
KG34	AACCCATCTCATAAATAACG
KG32	GTCCTTGAAGAAGATGGT
CaMV	CCACGTCTTCAAAGCAAGTG
PVIBR	TGTAGGGATAACAGGGTAAT
