# Supplementary material for: Plant X-tender: An extension of the AssemblX system for the assembly and expression of multigene constructs in plants
Source: PLoS One. 2018 Jan 4;13(1):e0190526. doi: 10.1371/journal.pone.0190526 (PMC5754074; doi:10.1371/journal.pone.0190526)
Supplement: S5 Table — (PDF) [file pone.0190526.s005.pdf]

S5 Table: Primers for sequencing and colony PCR.

	Backbone	Insert	Primers for sequencing	Primers for colony PCR	Length of the amplicon
Construction of Plant X-tender expression vectors	pCAMBIA_ASX	homology regions A0 and B0, I-*Sce*I and *Hin*dIII recognition sites and *ccd*B gene	M13F, M13R	M13F/M13R	896 bp
	pK7WG_ASX	homology regions A0 and B0, I-*Sce*I and *Hin*dIII recognition sites and *ccdB* gene	PVIBF, PVIBR8527	PVIBF/PVIBR	830 bp
	pH7WG_ASX	homology regions A0 and B0, I-*Sce*I and *Hin*dIII recognition sites and *ccd*B gene	PVIBF, PVIBR8527	PVIBF/PVIBR	830 bp
	pB7WG_ASX	homology regions A0 and B0, I-*Sce*I and *Hin*dIII recognition sites and *ccdB* gene	PVIBF, PVIBR8527	PVIBF/PVIBR	830 bp
Level 0 assembly	pL0A_0-R	p35S::H2BRFP_tNOS	FM176, FM218	FM176/FM218	3133 bp
	pL0A_0-1	p35S::H2BRFP_tNOS	FM176, FM218	FM176/FM218	2888 bp
	pL0A_1-R	pNOS::ECFP_t35S	FM176, FM218	FM176/FM218	2038 bp
Level 1 assembly	pL1A-hc / pL1A-lc (A0/AR)	p35S::H2BRFP_tNOS	FM182, FM131	KG022/FM182	773 bp
	pL1A-hc / pL1A-lc (A0/AR)	p35S::H2BRFP_tNOS + pNOS::ECFP_t35S	FM182, FM131, KG032	KG022/FM182, FM1113/FM032	773 bp, 702 bp
Plant X-tender expression vectors assembly and *A. tumefaciens* transformation	pCAMBIA_ASX	p35S::H2BRFP_tNOS	M13F, M13R	M13R/FM032	2854 bp
	pCAMBIA_ASX	p35S::H2BRFP_tNOS + pNOS::ECFP_t35S	M13F, M13R, KG032	M13R/KG022	716 bp
	pK7WG_ASX	p35S::H2BRFP_tNOS	PVIBF, PVIBR8527	PVIBF/FM032	2807 bp
	pH7WG_ASX	p35S::H2BRFP_tNOS	PVIBF, PVIBR8527	PVIBF/FM032	2807 bp
	pB7WG_ASX	p35S::H2BRFP_tNOS	PVIBF, PVIBR8527	PVIBF/FM032	2807 bp
