# Supplementary material for: Plant X-tender: An extension of the AssemblX system for the assembly and expression of multigene constructs in plants
Source: PLoS One. 2018 Jan 4;13(1):e0190526. doi: 10.1371/journal.pone.0190526 (PMC5754074; doi:10.1371/journal.pone.0190526)
Supplement: S6 Table — Expression cassette p35S::H2BRFP_tNOS (insert 1) and multigene construct p35S::H2BRFP_tNOS + pNOS::CFP_t35S (insert 2) were separately assembled into pCAMBIA_ASX to determine optimal conditions for insert cloning into the expression vectors. Bottom two rows represent a negative control (the plasmid backbone without the insert). m (ng): amount of pCAMBIA_ASX expression vector, AM: assembly method, SLiCE: Seamless ligation cloning extract cloning method, HiFi: HiFi DNA assembly method, MR: molar ratio between the plasmid backbone and the insert, TM: transformation method, E. coli: competent E. coli used for transformation, HM: homemade TOP10 chemically competent E.coli, HM: homemade TOP10 electrocompetent E.coli, C: commercial TOP10 chemically competent E.coli, T (μl): volume of assembly mixture used for transformation, number of colonies: the number of colonies grown after overnight incubation, cloning efficiency: the ratio between the number of clones with the correct insert length confirmed by colony PCR and the number of colonies subjected to colony PCR. (PDF) [file pone.0190526.s006.pdf]

S6 Table: Optimization of assembly and transformation methods for the insert cloning into Plant X-tender expression vectors. Expression cassette p35S::H2BRFP_tNOS (insert 1) and multigene construct p35S::H2BRFP_tNOS + pNOS::CFP_t35S (insert 2) were separately assembled into pCAMBIA_ASX to determine optimal conditions for insert cloning into the expression vectors. Bottom two rows represent a negative control (the plasmid backbone without the insert). m (ng): amount of pCAMBIA_ASX expression vector, AM: assembly method, SLiCE: Seamless ligation cloning extract cloning method, HiFi: HiFi DNA assembly method, MR: molar ratio between the plasmid backbone and the insert, TM: transformation method, *E. coli*: competent *E. coli* used for transformation, HM: homemade TOP10 chemically competent or electrocompetent *E.coli*, C: commercial TOP10 chemically competent *E.coli*, T (µl): volume of assembly mixture used for transformation, number of colonies: the number of colonies grown after overnight incubation, cloning efficiency: the ratio between the number of clones with the correct insert length confirmed by colony PCR and the number of colonies subjected to colony PCR.

insert	AM	m (ng)	MR	TM	*E. coli*	T (µl)	number of colonies	cloning efficiency
insert 1	SLiCE	50	1:10	electroporation	HM	2	167	3/6
	SLiCE	50	1:10	heat shock	HM	2	0	0/0
	SLiCE	50	1:10	heat shock	C	2	395	6/6
	SLiCE	50	1:2	electroporation	HM	2	15	5/5
	SLiCE	50	1:2	heat shock	HM	2	0	0/0
	SLiCE	50	1:2	heat shock	C	2	109	6/6
	SLiCE	150	1:2	electroporation	HM	2	107	6/6
	SLiCE	150	1:2	heat shock	HM	2	0	0/0
	SLiCE	150	1:2	heat shock	C	2	132	6/6
	HiFi	50	1:2	electroporation	HM	2	0	0/0
	HiFi	50	1:2	heat shock	C	2	0	0/0
	HiFi	50	1:2	heat shock	C	2	0	0/0
insert 2	SLiCE	50	1:10	electroporation	HM	1.5	41	6/11
	SLiCE	50	1:10	heat shock	HM	2	0	0/0
	SLiCE	50	1:10	heat shock	HM	3	1	0/1
	SLiCE	50	1:10	heat shock	C	2	108	8/10
	SLiCE	150	1:5	electroporation	HM	1.5	0	0/0
	SLiCE	150	1:5	heat shock	HM	2	0	0/0
	SLiCE	150	1:5	heat shock	HM	3	0	0/0
	SLiCE	150	1:5	heat shock	C	2	1	0/1
	SLiCE	150	1:2	electroporation	HM	1.5	0	0/0
	SLiCE	150	1:2	heat shock	HM	2	3	3/3
	SLiCE	150	1:2	heat shock	HM	3	1	1/1
	SLiCE	150	1:2	heat shock	C	2	880	6/10
	HiFi	50	1:5	electroporation	HM	2	0	0/0
	HiFi	50	1:5	heat shock	C	2	8	5/7
/	SLiCE	50	/	electroporation	HM	2	0	0/0
	SLiCE	50	/	heat shock	HM	2	0	0/0
